# Not all carbapenem-resistant Pseudomonas aeruginosa strains are alike: tailoring antibiotic therapy based on resistance mechanisms

**DOI:** 10.1097/QCO.0000000000001044

**Published:** 2024-09-18

**Authors:** Marco Falcone, Valentina Galfo, Giusy Tiseo

**Affiliations:** Infectious Diseases Unit, Department of Clinical and Experimental Medicine, Azienda Ospedaliero Universitaria Pisana, University of Pisa, Pisa, Italy

**Keywords:** carbapenem-resistant *Pseudomonas aeruginosa*, difficult-to-treat *Pseudomonas aeruginosa*, difficult-to-treat resistance, Guiana extended-spectrum, *Pseudomonas aeruginosa*

## Abstract

**Purpose of review:**

To correlate the resistance mechanisms and the susceptibility to new antibiotics in *Pseudomonas aeruginosa*.

**Recent findings:**

Definition of antibiotic resistance in *Pseudomonas aeruginosa* is still debated. Carbapenem-resistant *Pseudomonas aeruginosa* (CRPA) and difficult-to-treat resistant *Pseudomonas aeruginosa* (DTR-PA) are used but which of them better correlate with the risk of mortality remains debated. Mechanisms underlying resistance in *Pseudomonas aeruginosa* are complex and may be combined, resulting in unpredictable phenotype and cross-resistance. Thus, not all CRPA are alike and tailoring antibiotic therapy on resistance mechanisms is challenging.

**Summary:**

Current guidelines recommend the use of new antipseudomonal agents for CRPA or DTR-PA infections but they don’t provide specific information on how tailoring antibiotic therapy on underlying resistance mechanisms. This review may be useful to understand which mechanisms are involved in CRPA and may have practical implications helping clinicians to select an appropriate antibiotic regimen. Several antibiotics are now available for *Pseudomonas aeruginosa* but their rational use is important to avoid development of future resistance. The knowledge of local epidemiology and most common resistance mechanisms may guide empirical therapy, but targeted antibiotic therapy should be re-evaluated as soon as susceptibility testing profile is available and selected according to *Pseudomonas aeruginosa* phenotype.

## INTRODUCTION

*Pseudomonas aeruginosa* is a common cause of nosocomial infections, being responsible for about 7% of all health-care associated infections [1]. It can be responsible for infections in specific patients’ populations, including subjects with cystic fibrosis, noncystic fibrosis bronchiectasis, hematological disease, burn, and may be associated with high risk of mortality in these cohorts. Moreover, antimicrobial resistance in *Pseudomonas aeruginosa* remains a serious health threat because multiple mechanisms may be involved and further complicate the management of these infections [2]. Data from the European network EARS-Net highlight that in 2021 the 18.7% of *Pseudomonas aeruginosa* isolates were resistant to fluoroquinolones, 18.7% to piperacillin-tazobactam, 18.1% to carbapenems, 15.8% to ceftazidime and 8.9% to aminoglycosides [3]. Antimicrobial resistance mechanisms in *Pseudomonas aeruginosa* are very complex. Carbapenem resistance in Enterobacterales is usually mediated by enzymatic mechanisms, represented by the production of different types of carbapenemases [4–7] and depicts cases with very limited treatment options [8–12]. This could not be the case of carbapenem-resistant *Pseudomonas aeruginosa* (CRPA): they may be characterized by nonenzymatic resistance mechanisms and, despite the resistance to carbapenems, these isolates may have preserved susceptibility to other β-lactams, cephalosporins or fluoroquinolones [3,13^▪▪^]. In 2018, the definition of difficult-to-treat resistance (DTR) has been introduced to better categorize resistance in *Pseudomonas aeruginosa*[14]. DTR is defined as the presence of resistance to piperacillin-tazobactam, ceftazidime, cefepime, aztreonam, meropenem, imipenem, and fluoroquinolones [15].

Both the definitions of CRPA and DTR-PA pose clinical challenges and it's not clear which of the two definitions better correlate with the risk of mortality. A recent study demonstrated that CRPA are associated with a 30-day mortality of 32.8% and an attributable mortality of 19% [16^▪^]. A literature review empathized that DTR definition seems to better identify cases associated with highest risk of mortality compared to other resistance categories, including CRPA [13^▪▪^].

The recent availability of new antibiotics for CRPA and DTR-PA highlights the need for a comprehensive review of mechanisms underlying resistance in *Pseudomonas aeruginosa*. Currently available guidelines recommend the use of these novel antipseudomonal agents in patients with infections caused by CRPA or DTR-PA [15,17,18] but they don’t provide specific information on how tailoring antibiotic therapy based on underlying resistance mechanisms. Moreover, despite rare, resistance to new antibiotics may occur complicating the choice of the best antibiotic regimen in these patients. The aim of this review is to correlate the heterogeneous mechanisms conferring resistance in *Pseudomonas aeruginosa* and the susceptibility to new antipseudomonal antibiotics, providing some considerations useful in the clinical practice to select an appropriate antibiotic treatment in CRPA infections. 

**Box 1:**
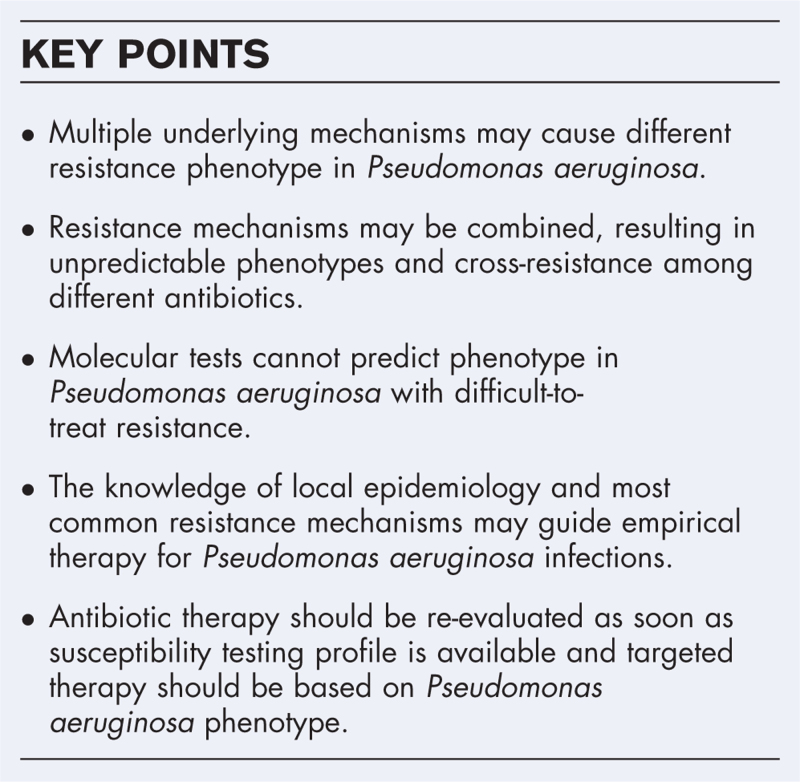
no caption available

### Literature search

A PubMed search with the following searching terms (*Pseudomonas aeruginosa*) AND (resistance mechanisms) AND (ceftolozane-tazobactam OR ceftazidime-avibactam OR meropenem-vaborbactam OR imipenem-relebactam OR cefepime-zidebactam OR cefepime-taniborbactam OR cefiderocol OR aztreonam-avibactam) AND (carbapenem OR beta lactams OR fluoroquinolones) was carried out, including articles published before 30 April 2024.

Some key points emerged from the literature review:

(1) multiple complex mechanisms can be responsible for resistance to carbapenems, new β-lactam/β-lactamase inhibitors (BLBLIs) and cefiderocol;
(2) mechanisms conferring resistance in *Pseudomonas aeruginosa* may be classified in: mutations in porins and efflux systems, enzymatic mechanisms (β-lactamases and carbapenemases), mutations in siderophores and altered iron transport;
(3) mechanisms conferring resistance in *Pseudomonas aeruginosa* may be combined, resulting in unpredictable phenotypes and cross-resistance among different antibiotics, further reducing the availability of active therapies;
(4) tailoring antibiotic therapy based on resistance mechanisms may be complex in CRPA and cannot be done without taking into account the microbiological phenotype.

### Mutational mechanisms of resistance: porins and efflux systems

Regulation of membrane permeability through porins and efflux pumps plays a major role in determining antibiotic resistance among *Pseudomonas aeruginosa* isolates. Inactivation or reduced expression of membrane porins, especially OprD, represents the most common mechanism conferring carbapenem resistance in CRPA [19]. OprD is used by the cell for amino acids uptake but it is also responsible of carbapenem internalization in bacterial cells [19]. Its inactivation can be caused by different mechanisms, including nonsense [20] and missense mutations [21], while under-regulation can be triggered by the excess of some metals including zinc, copper, and cadmium [22]. OprD mutations are associated with resistance to several antibiotics. Isolated alteration of OprD expression or activity results in resistance to imipenem and, to a less extent, meropenem [19]. In strains with isolated OprD inactivation relebactam seems to reduce the MICs of imipenem [23]. However, more recent data by Gomis-Font and collaborators showed that OprD inactivation represents the first step in resistance development to both imipenem and imipenem/relebactam [24]. OprD inactivation, especially when combined with other resistance mechanisms, such as efflux pumps overexpression, may cause resistance to ceftazidime/avibactam and ceftolozane/tazobactam [25,26^▪▪^].

The second mutational mechanism by frequency is represented by overexpression or mutation of efflux pumps, such as MexAB-OprM, MexXY, and MexST responsible for resistance to most β-lactams and carbapenems [27]. Overexpression of MexAB-OprM and MexXY-OprM represents mechanisms responsible for fluoroquinolone resistance, combined with mutations or increased expression of DNA gyrase and DNA topoisomerase [27,28]. Many efflux pumps are able to extrude β-lactamase inhibitors, playing a major role in resistance to new BLBLIs. In particular, MexAB-OprM and MexXY overexpression can impact *Pseudomonas aeruginosa* susceptibility to cefepime/zidebactam, cefepime/taniborbactam, ceftazidime/avibactam [27,29–31]. On the opposite ceftolozane is not a good substrate for the efflux pumps of *Pseudomonas aeruginosa*, and its activity is not compromised by upregulated Mex systems [27,32–34]. MeXAB-OprM overexpression, but not MexXY, also influences the susceptibility to the novel combination aztreonam/avibactam [27]. Finally, contrasting results emerged about the role of MeXAB-OprM and MexXY in resistance to imipenem/relebactam and cefiderocol [24,26^▪▪^,^35^–^38^].

Efflux pumps and membrane proteins genes may be regulated by the same gene: a significant example is represented by mutations of the MexEF-OprN regulators that also determines reduction of OprD expression [39].

The presence of porin loss and mutations in efflux pumps may not affect susceptibility to cefiderocol, due to its distinctive mechanism to gain entry into bacterial cells, mediated via iron-transport channels. In fact, a recent study by Shields and collaborators, demonstrates that resistance to traditional β-lactams (including ceftolozane/tazobactam, ceftazidime/avibactam and imipenem/relebactam), attributed to mutations in ampD, mexAB-oprM operon, and mexEF-oprN efflux operons, doesn’t impact the *in vitro* activity of cefiderocol [26^▪▪^]. Conversely, other authors hypothesized that overexpression of the mexAB-oprM multidrug efflux pump genes may contribute to cefiderocol resistance in *Pseudomonas aeruginosa* strains [35,40].

### Enzymatic mechanisms: β-lactamases and carbapenemases

Enzymatic mechanisms responsible for resistance in *Pseudomonas aeruginosa* are represented by *ampC* mutations [pseudomonal AmpC overexpression or AmpC variants, also called *Pseudomonas*-derived cephalosporinases (PDC)] and carbapenemases production.

Chromosomal constitutive (low expression) AmpC has been detected in up to 20% of *P. aeruginosa* isolates [41], representing the most frequent enzyme determining resistance to classical antipseudomonal penicillin, cephalosporins and monobactams. Ceftolozane/tazobactam and ceftazidime/avibactam are usually stable against induced or de-repressed AmpC. However, in the presence of other resistance mechanisms, AmpC (or PDC) induction and derepression may also result in resistance to new BLBLIs and carbapenems. AmpC hyperexpression is regulated by a large number of genes, such as those encoding for nonessential binding protein (PBP). PBP4 or PBP5 and PBP7 have been found to be the most frequent cause of derepressed *ampC* with subsequent BLBLIs resistance [42]. PBP, such as PBP5, may also have β-lactamase activity [43]. However, the role of PBPs in determining resistance to new molecules is under evaluation. Recent data suggest the involvement of PBP2 mutations in resistance to zidebactam [43] and cefiderocol [44], while PBP3 has been associated both to cefiderocol [31,44,45] and all new BLBLIs [27,46]. PDC variants, characterized by mutations of AmpC catalytic center, can determine resistance to ceftolozane/tazobactam and ceftazidime/avibactam [25,47,48].

The prevalence and the types of carbapenemases in *Pseudomonas aeruginosa* greatly varies in different regions, fact that underline the importance of the knowledge of local epidemiology. It ranges from 2% in the United States of America to 30% in Europe to more than 50% in South America, Australia and Singapore [49,50^▪▪^]. In Europe the main resistance mechanisms are represented by Verona integron-encoded metallo-β-lactamase (VIM) and Guiana extended-spectrum beta-lactamase (GES) [47,49], while *Klebsiella pneumoniae* carbapenemase (KPC) and metallo-β-lactamases (MBL) such as VIM represent the main resistance mechanism in North and South America and China; IMP and New Delhi metallo-β-lactamase (NDM) represent the main mechanisms in Middle East, Australia and Singapore [50^▪▪^]. GES is uncommon in North America, but in Europe its prevalence reaches 37% among CRPA, with GES-5 as the predominant variant [47,49]. GES production in *Pseudomonas aeruginosa* strains is important because it's generally associated with a peculiar phenotype: it confers resistance to carbapenems and ceftolozane/tazobactam, but not to ceftazidime/avibactam [2,47,49,51–53] and cefepime/taniborbactam [54]. Imipenem/relebactam remains active in the 83% of GES isolates [55], but it loses activity in isolates that produce GES-5 and KPC-2 [56].

The production of MBL causes resistance to several antibiotics, including carbapenems, ceftolozane/tazobactam, ceftazidime/avibactam, meropenem/vaborbactam, and imipenem/relebactam [25,57,58]. In these cases, cefiderocol [59,60] remain active against the majority of MBL-producing *Pseudomonas aeruginosa* isolates and may represent a therapeutic option. Moreover, the new combinations aztreonam/avibactam [61,62], cefepime/taniborbactam and cefepime/zidebactam [57,63] may also retain activity against MBL-producers.

Finally, an increasingly important role is played by OXA-10 β-lactamases. Even if a weak carbapenemase activity was reported, many OXA variants able to hydrolyze carbapenems have been described (OXA-40, OXA-198, OXA-655, and OXA-656) [64]. Some of them can be responsible for resistance against new molecules, including ceftolozane/tazobactam, ceftazidime/avibactam and cefiderocol [51,64,65].

### Siderophores and iron transport

Siderophores are iron-chelating molecules used by many species to facilitate iron transport and represent the main mechanism for cefiderocol entry into the bacterial cell. Two different siderophores, pyoverdine and pyochelin are produced by *Pseudomonas aeruginosa*[66]. More specifically, pyochelin sensitizes *Pseudomonas aeruginosa* to cefiderocol killing while pyoverdine displaces iron from cefiderocol [66].

Alterations in iron uptake [45], by siderophore mutations [35,67] and increased pyoverdine production [67], have been recently demonstrated to determine isolated cefiderocol resistance. Of note, high pyoverdine production by resistant strains also determines protections of susceptible isolates through a cross-protection mechanism [66].

### Combined mechanisms of resistance and cross resistance in *Pseudomonas aeruginosa*

The presence of combined mechanisms of resistance in *Pseudomonas aeruginosa* may lead to cross-resistance among different antibiotics. One of the most important cross resistance impacting the clinical practice is represented by ceftolozane/tazobactam plus ceftazidime/avibactam resistance. This phenomenon may be caused by mutations in the AmpC enzyme omega loop [25] or may occur in isolates that produce MBL [25], KPC, GES-1 and GES-15 [68,69]. A retrospective case-control study reported that 24.3% of *Pseudomonas aeruginosa* isolates were resistant to both ceftolozane/tazobactam and ceftazidime/avibactam [70]. Of importance, patients colonized or infected with these resistant strains had higher exposure to ceftolozane/tazobactam and cephalosporins compared to those with susceptible isolates [70].

Cross-resistance between ceftolozane/tazobactam and cefiderocol has been also reported. Shields *et al.* found it in the 21% of MDR *Pseudomonas aeruginosa* isolates [26^▪▪^]. It has been hypothesized that the presence of mutations in *ampC* and *tonB*-dependent receptors may be involved in these cases [26^▪▪^]. Conversely, cross resistance between cefiderocol and ceftazidime/avibactam or imipenem/relebactam was not documented [26^▪▪^].

The presence of other cross resistances may occur when additional resistance mechanisms are involved.

### Tailoring antibiotic therapy based on resistance mechanisms: is it possible for carbapenem-resistant *Pseudomonas aeruginosa*?

All the above-described mechanisms highlight the complexity of antibiotic resistance in *Pseudomonas aeruginosa*. Not all CRPA are alike because underlying mechanisms may be different and overlapping. Unlike CRE, in which the molecular resistance mechanism (genotype) usually predicts the susceptibility pattern (phenotype), the genotype-phenotype correspondence is not true for CRPA. Rapid molecular tests that search for carbapenemases cannot exclude the presence of a CRPA isolate since nonenzymatic resistance mechanisms or production of GES cannot be detected by commercially available rapid tests. Thus, rapid molecular tests have limited applicability in the clinical practice in the case of CRPA or DTR-PA infections. However, the knowledge of resistance mechanisms may be of importance for a rational choice of new antibiotics.

**FIGURE 1:**
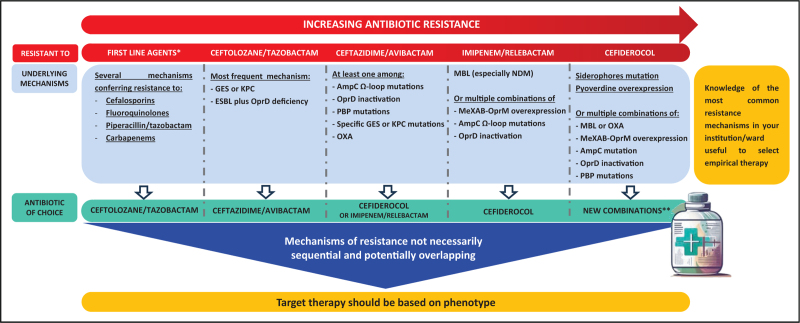
Scenarios of antibiotic resistance in *Pseudomonas aeruginosa* and underling mechanisms. ∗Resistance to: cefalosporins, fluoroquinolones, piperacillin/tazobactam, carbapenems (contemporary presence defines DTR-PA). ∗∗Cefepime/zidebactam, cefepime/taniborbactam, aztreonam/avibactam. MBL, metallo-β-lactamases; PBP, nonessential penicillin binding protein.

In this complex landscape, we tried to summarize the correlation between resistance mechanisms (genotype) and susceptibility to novel antipseudomonal agents (phenotype) and to provide some considerations useful for the clinical practice. Figure 1 shows different scenarios that clinicians may deal with. From left to right, this Figure describes more and more complex clinical situations. *Pseudomonas aeruginosa* isolates may be characterized by resistance to first-line antipseudomonal agents (cephalosporins, fluoroquinolones, piperacillin/tazobactam, carbapenems). In these cases (on the left of Fig. 1), ceftolozane/tazobactam should represent the first antibiotic option, when *in vitro* active [15,17,18,71]. In ceftolozane/tazobactam-resistant strains, ceftazidime/avibactam susceptibility ranges from 42.4% to 65% [51,72] and it reaches 100% if isolates harbor GES or KPC [2,51]. In these cases, ceftazidime/avibactam may be an appropriate therapeutic option [73,74]. However, cross resistance between ceftolozane/tazobactam and ceftazidime/avibactam (moving from the left to the right of Fig. 1), may represent a great challenge for clinicians, since limited options are available. In these cases, cefiderocol (or alternatively imipenem/relebactam when *in vitro* active) may be the most appropriate antibiotic option [25,26^▪▪^,^31^,^51^]. Cefiderocol may also be a therapeutic choice against *Pseudomonas aeruginosa* isolates that produce MBL [59,75,76]. A recent large retrospective, observational study (PERSEUS) conducted in patients with infections by Gram negative bacilli, mainly represented by *Pseudomonas aeruginosa*, treated with cefiderocol demonstrated positive outcomes [77]. In this study, among patients with infections by ceftolozane/tazobactam- and ceftazidime/avibactam resistant *Pseudomonas aeruginosa*, clinical success rates were 89.3% and 86.5%, respectively [77]. Further studies are warranted to evaluate whether monotherapy or combination therapy should be preferred.

Finally, when multiple mechanisms are combined (over-expression or mutation of AmpC, overexpression of MeXAB-OprM, siderophores mutations, pyoverdine overexpression, OXA or MBL production), resistance to cefiderocol may occur. In these cases, novel combinations, including cefepime/taniborbactam, cefepime/zidebactam and aztreonam/avibactam, may represent future alternatives, remaining susceptible in most MBL-producing isolates [57,61–63].

The knowledge of the most common resistance mechanisms in a specific institution or ward of hospitalization may help clinicians to select an appropriate empirical therapy. However, considering that all the described resistance mechanisms may overlap each other, the targeted antibiotic regimen should be based on the susceptibility pattern of the *Pseudomonas aeruginosa* isolate together with clinical considerations about site of infection and patients’ characteristics. This is the reason why antibiotic therapy cannot be tailored only based on resistance mechanisms but need to be confirmed according to susceptibility profile.

## CONCLUSION

Complex mechanisms are responsible for different patterns of antibiotic resistance in *Pseudomonas aeruginosa*. These mechanisms may be combined, resulting in unpredictable phenotypes and cross-resistance among different antibiotics, further reducing the availability of active therapies. However, several antibiotic options are now available but their rational use is important to avoid development of future resistance. When *in vitro* active, traditional antipseudomonal agents (cefepime, ceftazidime, piperacillin/tazobactam or – in some selected cases – fluoroquinolones) should be preferred to spare the use of new antibiotics. However, when these options are not suitable according to susceptibility pattern of the isolate, patients’ characteristics and/or implementation of carbapenem-sparing strategies, new antibiotics (such as ceftolozane/tazobactam and ceftazidime/avibactam) remain appropriate therapeutic options. Due to its activity against multiple determinants of resistance, cefiderocol appears the most appropriate choice in cases of suspected or proven DTR-PA resistant to all other agents. Further drugs (cefepime/taniborbactam, cefepime/zidebactam and aztreonam/avibactam) will enlarge the antibiotic armamentarium against DTR-PA.

## Acknowledgements

*None.*

### Financial support and sponsorship

*None.*

### Conflicts of interest

*G.T. received honoraria by Shionogi for educational meetings. M.F. received unconditional grants from MSD and grants/or speaker honoraria from Angelini, Shionogi, Pfizer, Menarini, Gilead, TermoFisher and Nordic Pharma. The remaining authors have no conflicts of interest.*

## REFERENCES AND RECOMMENDED READING

Papers of particular interest, published within the annual period of review, have been highlighted as:

▪ of special interest
▪▪ of outstanding interest

## References

[1] Weiner LM Webb AK Limbago B. Antimicrobial-resistant pathogens associated with healthcare-associated infections: summary of data reported to the National Healthcare Safety Network at the Centers for Disease Control and Prevention, 2011–2014. Infect Control Hosp Epidemiol 2016; 37:1288–1301. 27573805 10.1017/ice.2016.174 PMC6857725

[2] Lee SY Gill CM Nicolau DP. Activity of novel β-lactam/β-lactamase inhibitor combinations against serine carbapenemase-producing carbapenem-resistant *Pseudomonas aeruginosa*. J Antimicrob Chemother 2023; 78:2795–2800. 37840005 10.1093/jac/dkad225 PMC10689909

[3] Antimicrobial resistance surveillance in Europe 2023–2021 data. Stockholm: European Centre for Disease Prevention and Control and World Health Organization; 2023.

[4] Falcone M Tiseo G Galfo V. Bloodstream infections in patients with rectal colonization by *Klebsiella pneumoniae* producing different type of carbapenemases: a prospective, cohort study (CHIMERA study). Clin Microbiol Infect 2022; 28:298.e1–298.e7. 10.1016/j.cmi.2021.06.031 34197935

[5] Falcone M Tiseo G Antonelli A. Clinical features and outcomes of bloodstream infections caused by New Delhi metallo-β-lactamase-producing enterobacterales during a regional outbreak. Open Forum Infect Dis 2020; 7:ofaa011. 32042848 10.1093/ofid/ofaa011 PMC7003035

[6] Falcone M Giordano C Barnini S. Extremely drug-resistant NDM-9-producing ST147 *Klebsiella pneumoniae* causing infections in Italy, May 2020. Euro Surveill 2020; 25:2001779. 33272354 10.2807/1560-7917.ES.2020.25.48.2001779 PMC7716400

[7] Falcone M Bassetti M Tiseo G. Time to appropriate antibiotic therapy is a predictor of outcome in patients with bloodstream infection caused by KPC-producing *Klebsiella pneumoniae*. Crit Care 2020; 24:29. 32000834 10.1186/s13054-020-2742-9 PMC6993311

[8] Falcone M Giordano C. Clinical features and outcomes of infections caused by metallo-β-lactamase-producing enterobacterales: a 3-year prospective study from an endemic area. Clin Infect Dis 2024; 78:1111–1119. 38036465 10.1093/cid/ciad725

[9] Tiseo G Galfo V Riccardi N. Real-world experience with meropenem/vaborbactam for the treatment of infections caused by ESBL-producing Enterobacterales and carbapenem-resistant *Klebsiella pneumoniae*. Eur J Clin Microbiol Infect Dis 2024; [Epub ahead of print]. 10.1007/s10096-024-04758-2 38376634

[10] Falcone M Daikos GL Tiseo G. Efficacy of ceftazidime-avibactam plus aztreonam in patients with bloodstream infections caused by metallo-β-lactamase-producing enterobacterales. Clin Infect Dis 2021; 72:1871–1878. 32427286 10.1093/cid/ciaa586

[11] Falcone M Menichetti F Cattaneo D. Pragmatic options for dose optimization of ceftazidime/avibactam with aztreonam in complex patients. J Antimicrob Chemother 2021; 76:1025–1031. 33378458 10.1093/jac/dkaa549 PMC8861369

[12] Falcone M Mezzatesta ML Perilli M. Infections with VIM-1 metallo-{beta}-lactamase-producing *Enterobacter cloacae* and their correlation with clinical outcome. J Clin Microbiol 2009; 47:3514–3519. 19741074 10.1128/JCM.01193-09 PMC2772607

[13▪▪] Cosentino F Viale P Giannella M. MDR/XDR/PDR or DTR? Which definition best fits the resistance profile of *Pseudomonas aeruginosa*? Curr Opin Infect Dis 2023; 36:564–571. 37930070 10.1097/QCO.0000000000000966 PMC10836784

[14] Kadri SS Adjemian J Lai YL. Difficult-to-treat resistance in Gram-negative bacteremia at 173 US hospitals: retrospective cohort analysis of prevalence, predictors, and outcome of resistance to all first-line agents. Clin Infect Dis 2018; 67:1803–1814. 30052813 10.1093/cid/ciy378 PMC6260171

[15] Tamma PD Aitken SL Bonomo RA. Infectious Diseases Society of America 2023 guidance on the treatment of antimicrobial resistant Gram-negative infections. Clin Infect Dis 2023; ciad428 [Epub ahead of print]. 37463564 10.1093/cid/ciad428

[16▪] Falcone M Tiseo G Carbonara S. Mortality attributable to bloodstream infections caused by different carbapenem-resistant Gram-negative bacilli: results from a nationwide study in Italy (ALARICO Network). Clin Infect Dis 2023; 76:2059–2069. 36801828 10.1093/cid/ciad100

[17] Tiseo G Brigante G Giacobbe DR. Diagnosis and management of infections caused by multidrug-resistant bacteria: guideline endorsed by the Italian Society of Infection and Tropical Diseases (SIMIT), the Italian Society of Anti-Infective Therapy (SITA), the Italian Group for Antimicrobial. Int J Antimicrob Agents 2022; 60:106611. 35697179 10.1016/j.ijantimicag.2022.106611

[18] Paul M Carrara E Retamar P. European Society of Clinical Microbiology and Infectious Diseases (ESCMID) guidelines for the treatment of infections caused by multidrug-resistant Gram-negative bacilli (endorsed by European society of intensive care medicine). Clin Microbiol Infect 2022; 28:521–547. 34923128 10.1016/j.cmi.2021.11.025

[19] Pai H Kim J Kim J. Carbapenem resistance mechanisms in *Pseudomonas aeruginosa* clinical isolates. Antimicrob Agents Chemother 2001; 45:480–484. 11158744 10.1128/AAC.45.2.480-484.2001 PMC90316

[20] Sherrard LJ Wee BA Duplancic C. Emergence and impact of oprD mutations in *Pseudomonas aeruginosa* strains in cystic fibrosis. J Cyst Fibros 2022; 21:e35–e43. 33775602 10.1016/j.jcf.2021.03.007

[21] Richardot C Plésiat P Fournier D. Carbapenem resistance in cystic fibrosis strains of *Pseudomonas aeruginosa* as a result of amino acid substitutions in porin OprD. Int J Antimicrob Agents 2015; 45:529–532. 25735764 10.1016/j.ijantimicag.2014.12.029

[22] Ducret V Gonzalez MR Scrignari T Perron K. OprD repression upon metal treatment requires the RNA chaperone Hfq in *Pseudomonas aeruginosa*. Genes (Basel) 2016; 7:82. 27706108 10.3390/genes7100082 PMC5083921

[23] Livermore DM Warner M Mushtaq S. Activity of MK-7655 combined with imipenem against Enterobacteriaceae and *Pseudomonas aeruginosa*. J Antimicrob Chemother 2013; 68:2286–2290. 23696619 10.1093/jac/dkt178

[24] Gomis-Font MA Cabot G Sánchez-Diener I. In vitro dynamics and mechanisms of resistance development to imipenem and imipenem/relebactam in *Pseudomonas aeruginosa*. J Antimicrob Chemother 2020; 75:2508–2515. 32514525 10.1093/jac/dkaa206

[25] Ruedas-López A Alonso-García I Lasarte-Monterrubio C. Selection of AmpC β-lactamase variants and metallo-β-lactamases leading to ceftolozane/tazobactam and ceftazidime/avibactam resistance during treatment of MDR/XDR *Pseudomonas aeruginosa* infections. Antimicrob Agents Chemother 2022; 66:e0206721. 34930034 10.1128/aac.02067-21 PMC8846482

[26▪▪] Shields RK Kline EG Squires KM. In vitro activity of cefiderocol against *Pseudomonas aeruginosa* demonstrating evolved resistance to novel β-lactam/β-lactamase inhibitors. JAC Antimicrob Resist 2023; 5:dlad107. 37795425 10.1093/jacamr/dlad107 PMC10546814

[27] Oliver A Rojo-Molinero E Arca-Suarez J. *Pseudomonasaeruginosa* antimicrobial susceptibility profiles, resistance mechanisms and international clonal lineages: update from ESGARS-ESCMID/ISARPAE Group. Clin Microbiol Infect 2024; 30:469–480. 38160753 10.1016/j.cmi.2023.12.026

[28] Menon ND Somanath P Jossart J. Comparative molecular profiling of multidrug-resistant *Pseudomonas aeruginosa* identifies novel mutations in regional clinical isolates from South India. JAC Antimicrob Resist 2024; 6:dlae001. 38230352 10.1093/jacamr/dlae001 PMC10789591

[29] Barceló I. In vitro evolution of cefepime/zidebactam (WCK 5222) resistance in *Pseudomonas aeruginosa*: dynamics, mechanisms, fitness trade-off and impact on in vivo efficacy. J Antimicrob Chemother 2021; 76:2546–2557. 34219168 10.1093/jac/dkab213

[30] Sanz-García F Hernando-Amado S Martínez JL. Mutation-driven evolution of *Pseudomonas aeruginosa* in the presence of either ceftazidime or ceftazidime-avibactam. Antimicrob Agents Chemother 2018; 62:e01379–e1418. 30082283 10.1128/AAC.01379-18 PMC6153820

[31] Lasarte-Monterrubio C Fraile-Ribot PA Vázquez-Ucha JC. Activity of cefiderocol, imipenem/relebactam, cefepime/taniborbactam and cefepime/zidebactam against ceftolozane/tazobactam- and ceftazidime/avibactam-resistant *Pseudomonas aeruginosa*. J Antimicrob Chemother 2022; 77:2809–2815. 35904000 10.1093/jac/dkac241

[32] Castanheira M Kimbrough JH Lindley J. In vitro development of resistance against antipseudomonal agents: comparison of novel β-lactam/β-lactamase inhibitor combinations and other β-lactam agents. Antimicrob Agents Chemother 2024; 68:e0136323. 38526050 10.1128/aac.01363-23 PMC11064483

[33] Castanheira M Doyle TB Hubler CM. The plethora of resistance mechanisms in *Pseudomonas aeruginosa*: transcriptome analysis reveals a potential role of lipopolysaccharide pathway proteins to novel β-lactam/β-lactamase inhibitor combinations. J Glob Antimicrob Resist 2022; 31:72–79. 35931381 10.1016/j.jgar.2022.07.021

[34] Fournier D Carrière R Bour M. Mechanisms of resistance to ceftolozane/tazobactam in *Pseudomonas aeruginosa*: results of the GERPA multicenter study. Antimicrob Agents Chemother 2021; 65:e01117–e1120. 10.1128/AAC.01117-20 PMC7849014 33199392

[35] Sadek M Le Guern R Kipnis E. Progressive in vivo development of resistance to cefiderocol in *Pseudomonas aeruginosa*. Eur J Clin Microbiol Infect Dis 2023; 42:61–66. 36376766 10.1007/s10096-022-04526-0 PMC9816264

[36] Young K Painter RE Raghoobar SL. In vitro studies evaluating the activity of imipenem in combination with relebactam against *Pseudomonas aeruginosa*. BMC Microbiol 2019; 19:150. 31272373 10.1186/s12866-019-1522-7 PMC6610938

[37] Gomis-Font MA Cabot G López-Argüello S. Comparative analysis of in vitro dynamics and mechanisms of ceftolozane/tazobactam and imipenem/relebactam resistance development in *Pseudomonas aeruginosa* XDR high-risk clones. J Antimicrob Chemother 2022; 77:957–968. 35084040 10.1093/jac/dkab496

[38] Shields RK Stellfox ME Kline EG. Evolution of imipenem-relebactam resistance following treatment of multidrug-resistant *Pseudomonas aeruginosa* pneumonia. Clin Infect Dis 2022; 75:710–714. 35136967 10.1093/cid/ciac097 PMC9890448

[39] Li XZ Plésiat P Nikaido H. The challenge of efflux-mediated antibiotic resistance in Gram-negative bacteria. Clin Microbiol Rev 2015; 28:337–418. 25788514 10.1128/CMR.00117-14 PMC4402952

[40] Simner PJ Beisken S Bergman Y. Cefiderocol activity against clinical pseudomonas aeruginosa isolates exhibiting ceftolozane-tazobactam resistance. Open Forum Infect Dis 2021; 8:ofab311. 34262990 10.1093/ofid/ofab311 PMC8275882

[41] Horcajada JP Montero M Oliver A. Epidemiology and treatment of multidrug-resistant and extensively drug-resistant *Pseudomonas aeruginosa* infections. Clin Microbiol Rev 2019; 32:e00031-e19. 10.1128/CMR.00031-19 PMC6730496 31462403

[42] Ropy A Cabot G Sánchez-Diener I. Role of *Pseudomonas aeruginosa* low-molecular-mass penicillin-binding proteins in AmpC expression, β-lactam resistance, and peptidoglycan structure. Antimicrob Agents Chemother 2015; 59:3925–3934. 25896695 10.1128/AAC.05150-14 PMC4468666

[43] Smith JD Kumarasiri M Zhang W. Structural analysis of the role of *Pseudomonas aeruginosa* penicillin-binding protein 5 in β-lactam resistance. Antimicrob Agents Chemother 2013; 57:3137–3146. 23629710 10.1128/AAC.00505-13 PMC3697341

[44] López-Causapé C Maruri-Aransolo A Gomis-Font MA. Cefiderocol resistance genomics in sequential chronic *Pseudomonas aeruginosa* isolates from cystic fibrosis patients. Clin Microbiol Infect 2023; 29:538.e7–538.e13. 10.1016/j.cmi.2022.11.014 36435424

[45] Gomis-Font MA Sastre-Femenia MÀ Taltavull B. In vitro dynamics and mechanisms of cefiderocol resistance development in wild-type, mutator and XDR *Pseudomonas aeruginosa*. J Antimicrob Chemother 2023; 78:1785–1794. 37253034 10.1093/jac/dkad172

[46] Hernández-García M García-Castillo M Nieto-Torres M. Deciphering mechanisms affecting cefepime-taniborbactam in vitro activity in carbapenemase-producing Enterobacterales and carbapenem-resistant *Pseudomonas* spp. isolates recovered during a surveillance study in Spain. Eur J Clin Microbiol Infect Dis 2024; 43:279–296. 38041722 10.1007/s10096-023-04697-4

[47] Sastre-Femenia MÀ Fernández-Muñoz A Gomis-Font MA. *Pseudomonas aeruginosa* antibiotic susceptibility profiles, genomic epidemiology and resistance mechanisms: a nation-wide five-year time lapse analysis. Lancet Reg Health Eur 2023; 34:100736. 37753216 10.1016/j.lanepe.2023.100736 PMC10518487

[48] Alonso-García I Vázquez-Ucha JC Lasarte-Monterrubio C. Simultaneous and divergent evolution of resistance to cephalosporin/β-lactamase inhibitor combinations and imipenem/relebactam following ceftazidime/avibactam treatment of MDR *Pseudomonas aeruginosa* infections. J Antimicrob Chemother 2023; 78:1195–1200. 36918743 10.1093/jac/dkad062

[49] Gill CM Nicolau DP. ERACE-PA Global Study Group. Carbapenem-resistant *Pseudomonas aeruginosa*: an assessment of frequency of isolation from ICU versus non-ICU, phenotypic and genotypic profiles in a multinational population of hospitalized patients. Antimicrob Resist Infect Control 2022; 11:146. 36451179 10.1186/s13756-022-01187-8 PMC9710170

[50▪▪] Reyes J Komarow L Chen L. Global epidemiology and clinical outcomes of carbapenem-resistant *Pseudomonas aeruginosa* and associated carbapenemases (POP): a prospective cohort study. Lancet Microbe 2023; 4:e159–e170. 36774938 10.1016/S2666-5247(22)00329-9 PMC10016089

[51] Gill CM Nicolau DP. ERACE-PA Global Study Group. Phenotypic and genotypic profile of ceftolozane/tazobactam-nonsusceptible, carbapenem-resistant *Pseudomonas aeruginosa*. J Antimicrob Chemother 2022; 78:252–256. 36411249 10.1093/jac/dkac385 PMC9780534

[52] Poirel L Ortiz De La Rosa JM Kieffer N. Acquisition of extended-spectrum β-lactamase GES-6 leading to resistance to ceftolozane-tazobactam combination in *Pseudomonas aeruginosa*. Antimicrob Agents Chemother 2018; 63:e01809-e18. 10.1128/AAC.01809-18 PMC6325188 30323045

[53] Ortiz De La Rosa JM Nordmann P Poirel L. ESBLs and resistance to ceftazidime/avibactam and ceftolozane/tazobactam combinations in *Escherichia coli* and *Pseudomonas aeruginosa*. J Antimicrob Chemother 2019; 74:1934–1939. 31225611 10.1093/jac/dkz149

[54] Hamrick JC Docquier J-D Uehara T. VNRX-5133 (taniborbactam), a broad-spectrum inhibitor of serine- and metallo-β-lactamases, restores activity of cefepime in Enterobacterales and *Pseudomonas aeruginosa*. Antimicrob Agents Chemother 2020; 64:e01963-e19. 10.1128/AAC.01963-19 PMC7038240 31871094

[55] Ruiz VH Gill CM Nicolau DP. Assessing the in vivo impact of novel β-lactamase inhibitors on the efficacy of their partner β-lactams against serine carbapenemase-producing *Pseudomonas aeruginosa* using human-simulated exposures. J Antimicrob Chemother 2024; 79:546–551. 38217443 10.1093/jac/dkad412

[56] Li Y Fang L Dong M. blaKPC-2 overexpression and blaGES-5 carriage as major imipenem/relebactam resistance mechanisms in *Pseudomonas aeruginosa* high-risk clones ST463 and ST235, respectively, in China. Antimicrob Agents Chemother 2023; 67:e0067523. 37819082 10.1128/aac.00675-23 PMC10649045

[57] Karlowsky JA Hackel MA Wise MG. In vitro activity of cefepime-taniborbactam and comparators against clinical isolates of Gram-negative bacilli from 2018 to 2020: results from the Global Evaluation of Antimicrobial Resistance via Surveillance (GEARS) program. Antimicrob Agents Chemother 2023; 67:e0128122. 36541767 10.1128/aac.01281-22 PMC9872668

[58] Hernández-García M García-Castillo M Melo-Cristino J. In vitro activity of imipenem/relebactam against *Pseudomonas aeruginosa* isolates recovered from ICU patients in Spain and Portugal (SUPERIOR and STEP studies). J Antimicrob Chemother 2022; 77:3163–3172. 36059128 10.1093/jac/dkac298

[59] Gill CM Santini D Nicolau DP. ERACE-PA Global Study Group. In vitro activity of cefiderocol against a global collection of carbapenem-resistant *Pseudomonas aeruginosa* with a high level of carbapenemase diversity. J Antimicrob Chemother 2024; 79:412–416. 38153232 10.1093/jac/dkad396 PMC10832583

[60] Takemura M Wise MG Hackel MA. In vitro activity of cefiderocol against MBL-producing Gram-negative bacteria collected in North America and Europe in five consecutive annual multinational SIDERO-WT surveillance studies (2014–2019). J Antimicrob Chemother 2023; 78:2019–2027. 37390312 10.1093/jac/dkad200 PMC10393876

[61] Le Terrier C Nordmann P Poirel L. In vitro activity of aztreonam in combination with newly developed β-lactamase inhibitors against MDR Enterobacterales and *Pseudomonas aeruginosa* producing metallo-β-lactamases. J Antimicrob Chemother 2022; 78:101–107. 36308322 10.1093/jac/dkac360

[62] Karlowsky JA Kazmierczak KM de Jonge BLM. In vitro activity of aztreonam-avibactam against enterobacteriaceae and *Pseudomonas aeruginosa* isolated by clinical laboratories in 40 countries from 2012 to 2015. Antimicrob Agents Chemother 2017; 61:e00472-e17. 10.1128/AAC.00472-17 PMC5571336 28630192

[63] Mushtaq S Garello P Vickers A. Activity of cefepime/zidebactam (WCK 5222) against ’problem’ antibiotic-resistant Gram-negative bacteria sent to a national reference laboratory. J Antimicrob Chemother 2021; 76:1511-152. 10.1093/jac/dkab067 33760082

[64] Fratoni AJ Gethers ML Nicolau DP Kuti JL. Non-KPC attributes of newer β-lactamase/β-lactamase inhibitors. Part 1: enterobacterales and *Pseudomonas aeruginosa*. Clin Infect Dis 2024; ciae048. 10.1093/cid/ciae048 38306487

[65] Vuillemin X Da Silva M Bour M. Cefiderocol activity is compromised by acquired extended-spectrum oxacillinases in *Pseudomonas aeruginosa*. Int J Antimicrob Agents 2023; 62:106917. 37429451 10.1016/j.ijantimicag.2023.106917

[66] Galdino ACM Vaillancourt M Celedonio D. Siderophores promote cooperative interspecies and intraspecies cross-protection against antibiotics in vitro. Nat Microbiol 2024; 9:631–646. 38409256 10.1038/s41564-024-01601-4 PMC11239084

[67] Luscher A Moynié L Auguste PS. TonB-dependent receptor repertoire of *Pseudomonas aeruginosa* for uptake of siderophore-drug conjugates. Antimicrob Agents Chemother 2018; 62:e00097-18. 10.1128/AAC.00097-18 PMC5971595 29555629

[68] Mojica MF De La Cadena E García-Betancur JC. Molecular mechanisms of resistance to ceftazidime/avibactam in clinical isolates of enterobacterales and *Pseudomonas aeruginosa* in Latin American hospitals. mSphere 2023; 20:e0065122. 10.1128/msphere.00651-22 PMC10117078 36877058

[69] Fraile-Ribot PA Fernández J Gomis-Font MA. In vivo evolution of GES β-Lactamases driven by ceftazidime/avibactam treatment of *Pseudomonas aeruginosa* infections. Antimicrob Agents Chemother 2021; 65:e0098621. 34125593 10.1128/AAC.00986-21 PMC8370190

[70] Meschiari M Orlando G Kaleci S. Combined resistance to ceftolozane-tazobactam and ceftazidime-avibactam in extensively drug-resistant (XDR) and multidrug-resistant (MDR) *Pseudomonas aeruginosa*: resistance predictors and impact on clinical outcomes besides implications for antimicrobial stewardship programs. Antibiotics (Basel) 2021; 10:1224. 34680805 10.3390/antibiotics10101224 PMC8532599

[71] Meschiari M Asquier-Khati A Tiseo G. Treatment of infections caused by multidrug-resistant Gram-negative bacilli: a practical approach by the Italian (SIMIT) and French (SPILF) Societies of Infectious Diseases. Int J Antimicrob Agents 2024; 64:107186. 38688353 10.1016/j.ijantimicag.2024.107186

[72] Karlowsky JA Lob SH Bauer KA. Activity of ceftolozane/tazobactam, imipenem/relebactam and ceftazidime/avibactam against clinical Gram-negative isolates-SMART United States 2019-21. JAC Antimicrob Resist 2024; 6:dlad152. 38222461 10.1093/jacamr/dlad152 PMC10786191

[73] Garnacho-Montero J Sa-Borges M Sole-Violan J. Optimal management therapy for *Pseudomonas aeruginosa* ventilator-associated pneumonia: an observational, multicenter study comparing monotherapy with combination antibiotic therapy. Crit Care Med 2007; 35:1888–1895. 17581492 10.1097/01.CCM.0000275389.31974.22

[74] Almangour TA Ghonem L Alassiri D. Ceftolozane-tazobactam versus ceftazidime-avibactam for the treatment of infections caused by multidrug-resistant *Pseudomonas aeruginosa*: a multicenter cohort study. Antimicrob Agents Chemother 2023; 67:e0040523. 37404159 10.1128/aac.00405-23 PMC10433809

[75] Timsit JF Paul M Shields RK. Cefiderocol for the treatment of infections due to metallo-B-lactamase-producing pathogens in the CREDIBLE-CR and APEKS-NP Phase 3 randomized studies. Clin Infect Dis 2022; 75:1081–1084. 35148378 10.1093/cid/ciac078 PMC9522395

[76] Santerre Henriksen A Jeannot K Oliver A. In vitro activity of cefiderocol against European *Pseudomonas aeruginosa* and *Acinetobacter* spp., including isolates resistant to meropenem and recent β-lactam/β-lactamase inhibitor combinations. Microbiol Spectr 2024; 12:e0383623. 38483164 10.1128/spectrum.03836-23 PMC10986614

[77] Ramirez P, Merino E, Sarda J, *et al.* Real-world effectiveness and safety of cefiderocol in patients with Gram-negative bacterial infections in the early access program in Spain: results of the PERSEUS study. Abstract. ECCMID 2024.

